# Angiogenesis and Ovarian Cancer: What Potential Do Different Subtypes of Circulating Endothelial Cells Have for Clinical Application?

**DOI:** 10.3390/ijms25116283

**Published:** 2024-06-06

**Authors:** Du-Bois Asante, Domenico Tierno, Michael Woode, Bruna Scaggiante

**Affiliations:** 1 Department of Biomedical and Forensic Sciences, University of Cape Coast, Cape Coast P.O. Box CCLN 33, Ghana; duasante@ucc.edu.gh (D.-B.A.); michaelwoode44@gmail.com (M.W.); 2 Department of Medicine, Surgery and Health Sciences, University of Trieste, Strada di Fiume 447, I-34149 Trieste, Italy; tiernodomenico@gmail.com; 3 Department of Life Sciences, University of Trieste, Via Valerio 28, I-34127 Trieste, Italy

**Keywords:** angiogenesis, circulating endothelial cells, CECs, circulating endothelial progenitor cells, CEPCs, circulating tumour endothelial cells, CTECs, ovarian cancer

## Abstract

Ovarian cancer (OC) remains the most fatal disease of gynaecologic malignant tumours. The neovasculature in the tumour microenvironment principally comprises endothelial cells. Haematogenous cancer metastases are significantly impacted by tumour neovascularisation, which predominantly depends on the tumour-derived endothelial vasculogenesis. There is an urgent need for biomarkers for the diagnosis, prognosis and prediction of drug response. Endothelial cells play a key role in angiogenesis and other forms of tumour vascularisation. Subtypes of circulating endothelial cells may provide interesting non-invasive biomarkers of advanced OC that might have the potential to be included in clinical analysis for patients’ stratification and therapeutic management. In this review, we summarise the reported studies on circulating endothelial subtypes in OC, detailing their isolation methods as well as their potential diagnostic, prognostic, predictive and therapeutic utility for clinical application. We highlight key biomarkers for the identification of circulating endothelial cell subtypes and their targets for therapies and critically point out future challenges.

## 1. Introduction

Ovarian cancer (OC) is the leading cause of gynaecological cancer death worldwide [1]. More than 70% of women initially respond well to platinum–taxane-based chemotherapy at the advanced stages of the disease (stage III and IV), but unfortunately, most of them ultimately develop resistance, leading to treatment failure [2]. Thus, the identification of biomarkers that can aid in qualifying patients for clinical trials and help predict sustained responsiveness to treatment regimens in the advanced stages of the disease is of great importance.

Circulating rare cells encompass non-blood components in circulation, such as circulating tumour cells (CTCs) and circulating endothelial cells (CECs), the latter originating from either mature endothelial cells (ECs) or endothelial progenitor cells (EPCs) [3,4]. Several studies in different cancer types such as colorectal, breast, renal, pancreatic and non-small cell lung have highlighted the significance of CECs in tumour angiogenesis [5] and their presence in carcinoma clusters [6], which is crucial for tumour invasiveness and metastasis [7].

Liquid biopsy, a minimally invasive technique of detecting and analysing blood-borne biomarkers, has shown the potential of identifying markers of responsiveness in several cancer types in real-time [8]. Of critical note is the detection of rare cells and DNAs in the peripheral blood of OC patients that can help monitor disease response and also guide treatment decision [9]. CECs form a part of liquid biopsy and potentially allow for the serial sampling of blood from patients, ultimately providing a window of opportunity to predict responsiveness and longitudinal monitoring of individuals undergoing therapy [4]. In OC, for example, CEPCs levels significantly declined post-cytoreductive surgery and have also been shown to correlate with increased residual tumours [10]. Previous studies have also demonstrated that the CEPCs’ numbers significantly reduced in responders compared to non-responders, and more importantly, correlated with poor survival outcome [11]. However, in OC, very few studies have been performed to exploit the clinical outcome and predictiveness of response to markers of therapeutic importance on endothelial cells in the circulation of patients.

Herein, we review the reports on different circulating endothelial cell types in OC, i.e., circulating endothelial cells, circulating endothelial progenitor cells and circulating tumour endothelial cells, to explore their utility as biomarkers. We critically appraise the current evidence and focus on technical issues related to circulating endothelial cells detection and review results from clinical studies in OC for future potential applications.

## 2. Endothelial Cells and Neovascularisation Process

**Figure 1:**
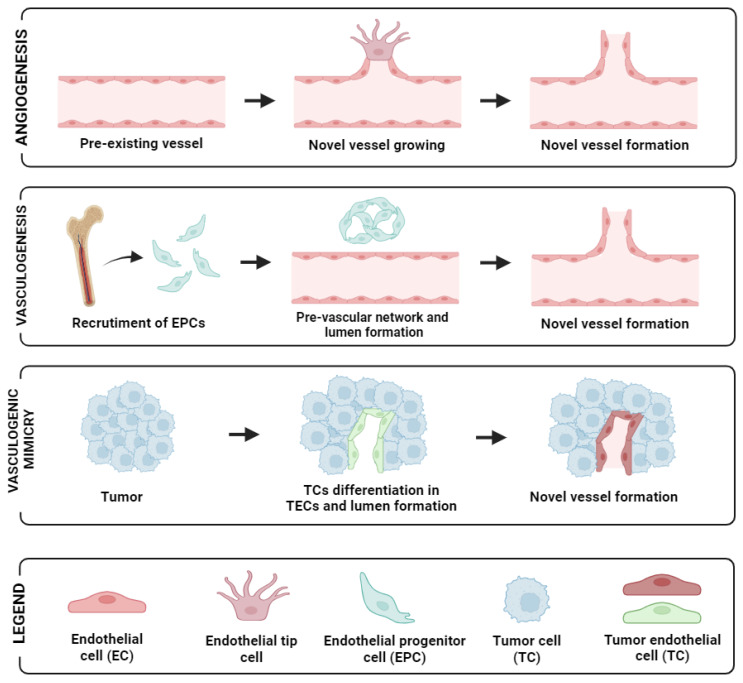
Schematic representation of the different neovascularisation processes: angiogenesis (up panel), vasculogenesis (mid-panel), and vasculogenic mimicry (low panel). Image created with biorender.

Angiogenesis is the process in which new blood vessels are generated from pre-existing vasculatures. In both physiological and pathological conditions, angiogenesis serves as a fundamental mechanism of vascular development and plays a pivotal role in enabling the rapid expansion of tumour cells via neovascularisation, ultimately facilitating metastasis. Of critical note is the presence of endothelial tip cells that are seen at the edges of sprout sites, leading and providing direction for neo-angiogenesis [12] (see Figure 1, first panel).

During tumour growth, tumours foster their own vascular network through alternative mechanisms, including vasculogenesis, vessel co-option, and vasculogenic mimicry [13]. In tumour vascular networks, angiogenesis plays a vital role in both the expansion and restructuring of dividing existing vessels branching to form daughter vessels [14]. Endothelial Progenitor Cells (EPCs) are also detectable in peripheral blood. In response to specific signals or cytokines, their concentration increases, which leads to their recruitment into the neovascular network of tumours [15].

Malignant cells initiate and enhance this process through releasing growth factors, cytokines, and chemokines, setting off a signalling cascade that shifts the balance towards the secretion of pro-angiogenic factors, thereby fostering the growth of blood vessels. Subsequently, these serve as chemo-attractants, aiding the recruitment of ECs to the site of neo-angiogenesis [16,17]. In contrast to the organised established mature structure of normal vasculatures, tumour vessels exhibit aberrant structural dynamics, vascular immaturity, and heightened permeability [18,19]. Eventually, these tumour vasculatures lose their polarisation and tightly packed arrangement, creating fenestrations for malignant cells to enter the bloodstream. This phenomenon culminates in aberrant vascularity, impaired vascular function, heightened permeability, augmented cellular motility, and elevated propensity for cancer metastasis [20].

In addition, another mechanism contributing to tumour vascularisation is vasculogenesis involving the recruitment of bone marrow-derived precursor cells, including endothelial and pericyte progenitor cells from circulation. These precursor cells subsequently differentiate into endothelial cells, leading to the de novo formation of vasculature within the tumour microenvironment [21]. In OC, this was demonstrated by Alvero et al. [22], who showed that stem-like OC cells possess the ability to function as tumour vascular progenitor cells. Specifically, stem-like OC cells expressing CD34+ and VE-cadherin+ markers were capable of generating xenograft tumours containing blood vessels lined with human CD34+ cells. Recently, another approach employing microvascular density has also been reported to have a significant association with clinical-pathological parameters in primary OC [23]. Moreover, vasculogenic mimicry (VM), the phenomenon where tumour cells mimic endothelial characteristics, has been reported in several cancer types including OC [24]. For instance, the more mesenchymal and invasive OC cell line SKOV3 demonstrated the capacity to form vascular channels. By silencing CD147 (matrix metalloproteinase inducer) in SKOV3, its vasculogenic characteristics exhibited an impaired ability to form vascular channels [25]. Similarly, CD177-positive tumours were found to correlate significantly with VM formation, as well as with various tumour characteristics and prognosis. Patients with CD177-positive tumours exhibited shorter survival outcomes compared to those with CD177-negative tumours [26]. A diagram of angiogenesis and the supporting mechanisms for neovascularisation in tumours is shown in Figure 1. Altogether, these observations suggest that angiogenesis enhances tumour growth and dissemination, and with multiple studies alluding to these facts in OC, this necessitates potential marker identification in a non-invasive fashion in peripheral blood to aid and predict responsiveness and prognosis in patients with this disease.

## 3. Circulating Endothelial Cell Subtypes

Circulating rare cells encompass circulating tumour cells (CTCs) and circulating endothelial cells (CECs), the latter originating from ECs within blood vessels entering circulation. Several studies have highlighted the significance of CECs in tumour angiogenesis and their presence in carcinoma clusters [6], which is crucial for tumour invasiveness and metastasis. In oncological studies, technically, CECs are heterogenous, having three different subtypes and being identified based on the differential biomarkers they express. They can either be terminally differentiated cells derived from blood vessels (matured circulating endothelial cells, or CECs) or bone marrow-derived circulating endothelial progenitor-like cells (CEPCs) that incorporate into new blood vessels [5]. The third is tumour-derived CECs, termed circulating tumour endothelial cells (CTECs), thought to be derived from the tumour microenvironment via vasculogenic mimicry or trans-differentiation [24]. The latter, CTECs, show cytogenetic irregularities such as aneuploidy and the ability to resist anchorage-dependent cell death [27,28]. Elevated levels of these CECs in peripheral blood of cancer patients compared to healthy controls have been reported [4,5]. Furthermore, increased CECs count in cancer patients has been demonstrated to be nearly normalised after tumour chemotherapy or surgical resection [29]. Therefore, these CECs might reflect the extent of tumour angiogenesis. CD31 is a common molecule among these diverse circulating endothelial subtypes [30]. During endothelial lineage differentiation, early circulating endothelial CD31+/CD34+/CD133+ progenitors (CEPCs) exhibit down-regulated CD133 expression and increased CD31 expression to mature into CD31+/CD34+/CD133− CEPCs, further developing into CD31+/CD146+ conventional CECs [5]. CD45 (white blood cell marker) is used as a negative selection marker. Notably, for the tumour-derived counterpart, CTECs and CD45−/CD31+, they are mostly identified via fluorescent in situ hybridisation (FISH) methods [31].

### 3.1. Circulating Endothelial Cells in OC

All three different subtypes of endothelial cells (CECs, CEPCs and CTECs) mentioned above have been identified in studies of OC patients’ blood samples (Table 1). Eleven studies [10,11,32,33,34,35,36,37,38,39,40] were present in the literature. Five studies identified CECs, seven identified CEPCs and only one study reported on CTECs (Table 1). Two of these studies [11,37] identified both CECs and CEPCs in their enriched peripheral blood samples. CD31 and 34 were the predominant markers for the detection of the different subtypes of endothelial cells. Other markers include VE-cadherin-negative CECs to distinguish them from CTC [35], VEGFR-2&3 and the von Willebrand factor. Differential markers used to distinguish CEPCs from CECs included CD133 and CD146 (Table 1).

### 3.2. Potential Biomarkers for the Detection of the CECs/CEPCs

CD31 is a common molecule among diverse CEC subtypes [30]. As mentioned above, early circulating endothelial CD31+/CD34+/CD133+ progenitors (CEPCs) exhibit down-regulated CD133 expression and increased CD31 expression to mature into CD31+/CD34+/CD133− CEPCs, further developing into CD31+/CD146+ conventional CECs [5,41]. Cheng et al. [33] introduced a novel strategy, single-cell enumeration iFISH (SE-iFISH), for the comprehensive detection and characterisation of aneuploid-circulating rare cells, including CTCs and CECs in patient blood. They identified chromosome 8 aneuploid CD31+ CECs in samples from both OC and benign ovarian tumour patients, with a subset expressing CD146 or CD34. These CD31+ CECs lacked CD133 and CD105 expression, indicating heterogeneity. Furthermore, a novel subtype lacking CD34, CD133, CD105, and CD146 was identified, comprising the majority of aneuploid CD31+ CECs. The study also demonstrated the concurrent detection of aneuploid CECs and CTCs exhibiting different epithelial-to-mesenchymal transition (EMT) statuses using SE-iFISH. Clinical validation confirmed the co-detection of vimentin, EpCAM, and CD31 on CECs and CTCs in a broader cohort of cancer patients. Conclusions from the studies mentioned highlight vimentin, EpCAM and CD31 as potential biomarkers for the detection of CTECs.

Additionally, biomarkers utilised for the detection of CEPCs across multiple studies include CD34, CD133, VEGFR-2, and VEGFR-3 [11,34,36,37,38,42].

In recent years, considerable attention has been directed towards programmed death ligand 1 (PD-L1) due to its significant role in maintaining an immunosuppressive tumour microenvironment by negatively modulating anti-tumour responses, leading to anergy or the exhaustion of programmed death receptor 1 (PD-1)-expressing T cells [43]. Numerous studies have illustrated the correlation of PD-L1 with tumours displaying a mesenchymal phenotype and its association with malignant progression [44]. The expression of PD-L1 on vascular ECs has garnered interest in the field of oncology [45]. Some evidence suggests the potential of combining this with anti-angiogenic therapy and immunotherapy for selected patients. For instance, PD-L1 expression on CECs from non-small cell lung cancer patients undergoing immunotherapy has been linked to favourable patient outcomes [46]. In the context of the above considerations, it is interesting to cite a recent study, in which the identification of CD31+ CECs expressing PD-L1 holds significance in OC, suggesting further investigation into future clinical trials focusing on combined anti-angiogenic and immunotherapies [40].

### 3.3. Isolation Platform and Detection Method

**Figure 2:**
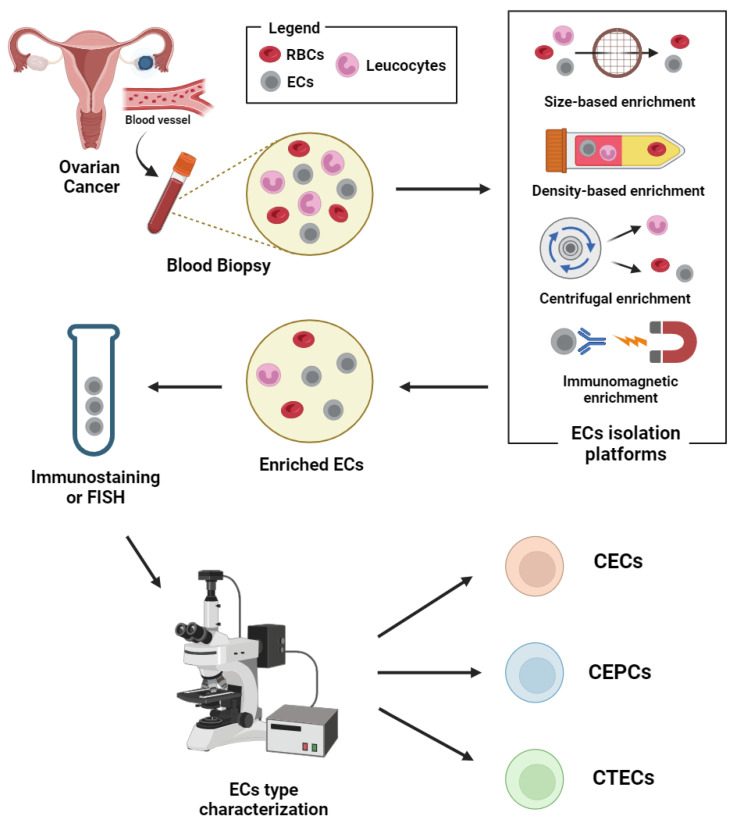
Schematic representation of circulating endothelial cells isolation and detection workflow. Abbreviations: CECs: circulating endothelial cells; CEPCs: circulating endothelial progenitor cells; CTECs: circulating tumour endothelial cells; ECs: endothelial cells; RBCs: red blood cells. Image created with biorender.

The ratio of ECs to other cells in the blood is extremely low. Thus, the isolation platforms help in the enrichment and aid in differentiating CECs/CEPCs/CTECs from haematopoietic cells in whole blood. Various methods were reported for the isolation of these rare cells; (1) physical properties of tumour cells such as density and size, (2) biological properties such as positive or negative label-dependent immunoaffinity enrichment targeting specific surface markers (Table 1) (Figure 2).

Isolation methods based on physical properties (label-free) include the use of micro-fluidic platforms such as Parsortix^TM^ and density gradient centrifugation. The immunomagnetic methods, though specific in targeting markers on the endothelial cells, do not comprehensively target the heterogenous subsets of endothelial-derived cells in the blood [47]. Thus, more markers may be needed for isolation, if the heterogenous subsets of those that are endothelial-derived are to be considered during the enrichment.

The immunofluorescence technique was the most used (seven out of eleven studies) to detect blood-borne endothelial cells after isolation in the OC studies. Though this technique is user friendly, including flow cytometry, the more tedious upstream genetic and molecular analysis of CECs from OC [33,48] may help identify the tumorgenicity of these cells classified as CTECs.

Overall, most studies did not report on the sensitivity and specificity of the assay used, which affects the comparison of the diagnostic performance of different platforms employed for the detection and analysis of these rare cells in OC. Therefore, the robustness and clinical validity of these techniques across different platforms beyond the initial proof of concept warrants further study using a larger sample size.

### 3.4. Potential of CECs/CEPCs/CTECs as Biomarkers in Ovarian Cancer

Multiple studies in various cancer types have persistently associated the reduction in CTECs or CECs with reduced tumour burden or favourable patients’ outcomes. For instance, CTECs decrease in number after operations in oesophageal and lung cancers, correlating with a reduction in tumour growth [49]. In a more recent study [31], the combined detection of CTCs and CTECs aided in predicting prognosis (overall survival) in patients with advanced lung cancer. Similarly, CEC numbers significantly declined after treatment in breast cancer and lymphoma patients [50].

In OC, circulating levels of VEGFR2+ bone marrow-derived CEPCs were shown to have clinical significance [10]. Their levels rapidly declined following cytoreductive surgery. Also, in the same study, CEPCs levels significantly increased in non-responsive OC patients undergoing treatment with chemotherapy and correlated with increased residual tumours. Higher levels of CEPCs in the study were also associated with advanced stage (III and IV) of the disease, compared with the early stages (I–II). Similarly, in a phase II clinical trial that sought to identify potential lead biomarker candidates for a response to combined Olaparib (a PARP inhibitor) and Cediranib (VEGFR1–3 inhibitor) in recurrent platinum-sensitive OC patients, the authors reported that there was a significant decrease in CEC numbers in the combined Olaparib and Cediranib cohort compared with the group that was treated with Olaparib alone (*p* < 0.05) [11]. This depicted a higher treatment efficacy in the combined treatment group than the Olaparib alone.

Evaluating the quantity of CECs, EPCs and their tumour counterpart, circulating tumour-associated (CTECs) in other cancer types have been reported to be associated with reduced tumour burden or favourable patients’ outcomes. Others have associated the responsiveness and patient’s outcome to potentially druggable markers such as PD-L1 and VEGF-R, etc., on the endothelial cells of cancer patients [4,51].

Furthermore, cancer patients (including those with ovarian cancer) with progressive disease had an average of 3.6-fold more CECs than healthy controls (*p* > 0.05). Those with stable disease, however, had CEC numbers equal to that of the healthy control group (*p* > 0.05) [32]. Thus, evaluating CEC numbers can be used to differentiate between progressive and stable disease states in OC patients. Similar results were reported in a previous study [34] using cervical and OC patients.

A very interesting finding using epithelial OC patients was reported by Qiu et al. [36]. In this study, there was a statistically significant correlation between CEPC levels and surgical staging of epithelial OC (*p* < 0.05). That is, CEPCs levels correlate with lymph node metastasis. Also, the level of CEPCs was significantly higher in OC patients compared with that of healthy control subjects.

Thus, CEPCs can be a surrogate marker to monitor progression and treatment response in late-stage and recurrent OC patients.

Of note, another study using OC patients [33] had no significant association comparing the levels of CTECs in the patients and benign counterparts. Though the counts of CTECs were reported to be higher in the OC group than in the benign group, the difference was not statistically significant (*p* < 0.05). This study used newly diagnosed individuals and thus could not evaluate CTECs numbers pre- and post-treatment, based on treatment response.

The main findings discussed in this section are summarised in Table 2.

## 4. Perspectives

Basically, in OC, CEC subtype analysis can give information on the tumour angiogenesis that might be relevant for patients’ management and treatment decision. This current review on OC CECs/CEPCs/CTECs in OC patients identified significant variability between studies, isolation platforms, detection methods and markers of detection. Cut-off values used for confirming endothelial cell positivity varied across different platforms, which could potentially have an impact on the derived results. Therefore, a more uniform approach to CECs/CEPCs/CTECs characterisation and definitions of positivity are needed to evaluate intra- and inter-laboratory reproducibility. Furthermore, though detection via immunocytochemistry has been the conventional way of identifying CECs/CEPCs in the peripheral blood of OC patients, the inclusion of upstream molecular analysis will help validate the tumourigenicity of these rare cells (CTECs). Only one study reported on CTECs in OC. Given that these subtypes are intrinsically involved in neo-angiogenesis and metastasis in the tumour microenvironment, clinical trials targeting and analysing these cells using large sample sizes for the longitudinal study of treatment response are warranted.

More applicable to clinical settings, the evaluation of CECs/CEPCs/CTECs as a potential biomarker for drug responsiveness will be best demonstrated using treatment regimes or therapies with known efficacy such as the anti-angiogenic agent bevacizumab. This allows for a fair assessment of these biomarkers’ diagnostic and prognostic utility. In the case of Schilder et al.’s [37] study in OC patients, Motesanib was used (the drug’s efficacy was still being evaluated); unfortunately, adverse events from the treatment brought about an early closure of the clinical trial. Thus, the diagnostic and prognostic utility of CEPCs and CECs was not further assessed.

A combined evaluation of CECs/CEPCs/CTECs and CTCs via the immunostaining of an OC patient’s blood would have been a great idea, as it is cost effective compared to molecular analysis. However, the number of fluorescent channels is mostly four and this does not allow for the identification of extra cell types and also prevents effective phenotypic characterisation of these rare cells when present. The advent of fluorescent staining, quenching and re-staining methods on rare circulating cells have allowed for extra fluorescent biomarker (nine or more) application on CTCs from patient’s blood [52]. This method can be employed not only for the co-detection of CTCs and CECs/CEPCs/CTECs but also for the effective phenotypic characterisation of these cells such as the expression of actionable markers, PD-L1, PD-1, etc., on these rare cells [52]. The advent of molecular-targeted therapies has revolutionised OC treatment and is now moving beyond conventional chemotherapy. For instance, over the last two decades, the use of immunotherapy has transformed the treatment of various cancer types [53]. Recently, the mostly used checkpoint inhibitors (ICIs), which include CTLA-4 and programmed cell death protein 1 (PD-1)/PD-L1 inhibitors acting through reversing the immunological signals from the immunosuppressive tumour microenvironment, have attracted significant attention in OC oncotherapeutics [54]. Similarly, anti-angiogenic therapies that block angiogenesis and thus prevent tumours from developing their own blood vessels have been extensively investigated for their efficacy in OC, and results are promising [55]. Combinatorial therapies that synergistically target different areas of the tumour microenvironment, such as the combined ICI and anti-angiogenic inhibitors, have shown promise in OC [56]. However, markers that can help predict responsiveness to combined therapy such as ICI and anti-angiogenic inhibitors are lacking.

**Figure 3:**
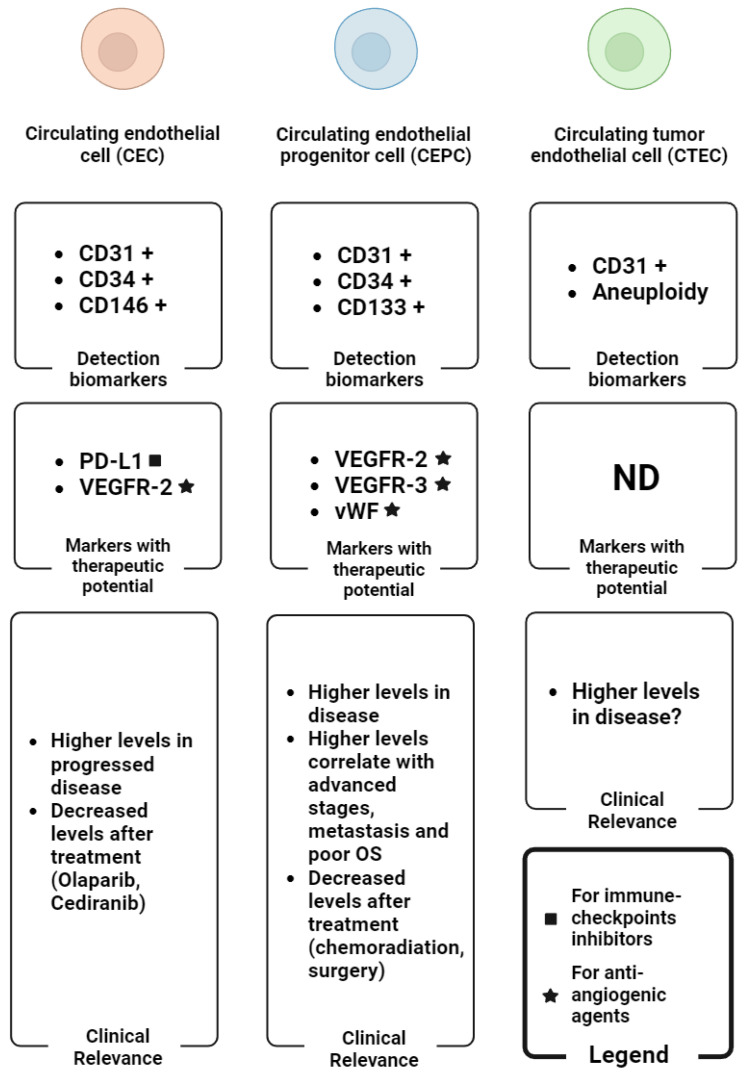
Graphical summary of the main biomarkers for the detection of CEC subtypes (detection biomarker boxes), the CEC subtypes-associated biomarkers with therapeutic potential (markers with therapeutic potential boxes) and the main clinical findings regarding the levels of CEC subtypes in OC (clinical relevance boxes). The plus symbol (+) indicates the expression of the corresponding biomarker in reference cells. Abbreviations: ND: not determined; OS: overall survival. Image created with biorender.

In OC studies, the evaluation of other markers of therapeutic importance such as PD-L1 in association with a patient’s outcome has not been elucidated. In a more recent preliminary study, Asante et al. [40] demonstrated the potential predictive utility of both PD-L1+ CTCs and CECs in OC patients for the first time. Hence, the identification of CD31+ cells expressing PD-L1 may be of clinical relevance and warrant further future studies on CECs in OC studies. Overall, the advanced stages have a higher tumour burden and thus can release an enormous number of CECs, CEPCs, and CTECs into circulation following treatment, but the numbers decline with time in the responders or the stable disease individuals. Progressive and non-responsive subjects may have continuous high levels of these rare cells in their peripheral blood. To highlight this potential, the specific biomarkers and the clinical values of the CEC subtypes in OC are summarised in Figure 3.

## 5. Conclusions

All in all, circulating endothelial cell types are attractive potential biomarkers for OC that could be useful to ensure the best clinical management and the right therapeutic choice. To achieve this goal, many technological challenges and clinical studies are needed in this field. The standardisation across different CECs/CEPCs/CTECs enrichment and detection platforms is an urgent need. Also, future studies in the endothelial cells (CECs/CEPCs/CTECs) in the peripheral blood are necessary to provide an in-depth insight into the mechanistic molecular role that these cells play in OC, its relationship with ICIs such as PD-L1, and more importantly, its association with survival outcomes. Of critical note is the low number of OC patients in most of the studies; this ultimately requires validation in larger cohorts. However, the studies discussed above reflect the great potential of CECs, CEPCs and CTECs in the treatment of OC and represent a promising starting point for their comprehensive analysis in the near future.

## Figures and Tables

**Table 1:** Studies that detected CECPs, CECs and CTECs in ovarian cancer.

Subtypes (Stage)	Isolation Platform	Method of Detection	Biomarkers Used for Detection	CECs/CEPCs/CTECs	Cit.
HGSOC (III-IV)	Parsortix (Microfluidics)	Immunofluerescence	CD31	CECs	[40]
/	Immunomagnetic	Immunostaining and fluorescence in situ hybridisation (iFISH)	CD31 aneuploidy	CTECs	[33]
HGSOC, clear cell carcinoma	Immunomagnetic cell surface target	Immunofluorescence	VE-cad	CECs	[35]
HGSOC (platinum sensitive)	Density gradient centrifugation	Flow cytometry	CD31, CD146 CD133	CECs, CEPCS	[11]
Serous and mucinous (IB, IIA, IIB, IIIC)	Density gradient centrifugation	Quadruple immunofluorescence	CD34, CD133	CEPCs	[34]
Serous, endometroid, and mucinous (FIGO I-IV) and clear cell	Density centrifugation	Flow cytometry	CD34	CEPCs	[36]
Serous, mucinous, and endometroid	Density gradient centrifugation	Immunofluorescence	CD31	CEPCs	[39]
Clear cell, endometroid, and serous	Immunomagnetic	Immunofluorescence	CD34, CD-133 CD-146	CEPCs, CECs	[37]
Serous, mucinous and endometrioid (I–IV)	Immunomagnetic	Flow cytometry, RT-PCR	CD34	CEPCs	[38]
Serous, mucinous and endometroid	Density gradient centrifugation	Immunofluorescence	CD31	CEPCs	[10]
/	Immunomagnetic	Immunofluorescence	CD31	CECs	[32]

Abbreviations: CECs: circulating endothelial cells; CEPCs: circulating endothelial progenitor cells; CTECs: circulating tumour endothelial cells.

**Table 2:** Main clinical findings from CECs, CEPCs, and CTECs in ovarian cancer studies.

Patients/ Control (n/n)	Stage	Circulating Endothelial Cell Types/Clinical Value	Markers of Therapeutic Importance	Main Clinical Findings	Cit.
P/HC(16/5)	I-IV	CECs Diagnostic, Predictive	PD-L1	Subsets of CD31+ in OC patients were PD-L1+/CK+/EpCAM+.	[40]
P/HC(20/36)	NR	CTECs Diagnostic	/	CTEC levels were higher in OC patients than in benign cases. However, the difference was not significant.	[33]
P(13)	NR	CECs and CEPCs Prognostic	/	Patients who received a combination of Olaparib and Cediranib had significant decrease in IL-8 concentration and CECs numbers, compared with patients who received Olaparib alone.	[11]
P/HC(14/14)	IB, IIA, IIB, IIIC	CEPCs Diagnostic, Prognostic	VEGFR-2	Patients who underwent chemoradiation therapy or surgery had a reduced frequency and number of CEPCs compared to pre-treatment values.	[34]
P/HC(54/31)	I–IV	CEPCs Diagnostic, Predictive	VEGFR-3	CEPC levels were higher in OC patients compared with healthy controls, and the increase in CEPC levels correlated with lymph node metastasis.	[36]
P(22)	NR	CECs and CEPCs /	vWF, VEGFR-2	CPEC/CEC levels were higher in second/third cycles of treatments compared to the first one. This could be due to the side-effects of Motesanib.	[37]
P/HC(22/15)	NR	CEPCs Diagnostic	VEGFR-2	CEPCs from OC patients showed increased expression of Id1 and MMP-2 compared to those from healthy controls. Id1 was involved in stimulation of angiogenesis, tumour proliferation and migration via PIK3CA/Akt and NF-kB/MMP2 pathway.	[39]
P/HC(42/25)	I–IV	CEPCs Diagnostic, Prognostic	VEGFR-2	CEPCs levels significantly increased in in OC patients compared to healthy control. Higher levels in stages III and IV compared to stages I and II. High CEPCs count correlated with poor overall survival.	[10]
P/HC(20/25)	NR	CEPCs Diagnostic, Predictive	vWF, VEGFR-2	CEPCs from OC patients showed an increased expression of Id1 and integrin α4 compared to those from healthy controls. Id1 mediated CEPCs mobilisation and recruitment. Inhibition of PI3K/Akt of cultured CEPCs from OC patients, down-regulated the expression of Id1 and integrin α4, inhibiting CEPCs mobilisation.	[38]
P/HC(95/46)	NR	CECs Diagnostic, Predictive	VEGFR-2	Compared to healthy cohort, OC patients with progressing disease exhibited an average of 3.6 times higher CECs. CECs levels in OC patients with stable disease were similar to those of healthy controls.	[32]

Abbreviations: B: benign; CECs: circulating endothelial cells; CEPCs: circulating endothelial progenitor cells; CTECs: circulating tumour endothelial cells; HC: healthy controls; OC: ovarian cancer; NR: nor reported; n: number; P: patients.
